# What captures women’s visual attention and what they remember in romantic encounters: eye-tracking metrics and self-reports

**DOI:** 10.3389/fpsyg.2026.1773619

**Published:** 2026-04-15

**Authors:** Oleksandra Romaniuk, Jörg Mühlhans, Ulrich Ansorge

**Affiliations:** 1 Department of English and American Studies, University of Vienna, Vienna, Austria; 2 MediaLab, Faculty of Philological and Cultural Studies, University of Vienna, Vienna, Austria; 3 Department of Cognition, Emotion, and Methods in Psychology, University of Vienna, Vienna, Austria

**Keywords:** attentional biases, dual-process model, eye-tracking, introspective awareness, mate selection, romantic attraction cues, visual salience

## Abstract

**Introduction:**

Visual cues play a central role in romantic attraction, yet individuals may not accurately recall what captured their attention. Drawing on dual-process models of cognition, this study examined the correspondence between measured gaze behavior and retrospective self-reported salience in women’s romantic perception.

**Methods:**

Sixty-eight women viewed 10 romantic scenes portraying heterosexual couples while their eye movements were recorded. Five areas of interest (AOIs)—female face, male face, female body, male body, and background—were analyzed across key eye-tracking metrics: total dwell time, average fixation duration, fixation count, and first fixations. Participants then ranked these AOIs by perceived salience and reported up to three visual attraction cues (e.g., smile, hands, legs) per AOI.

**Results:**

Women’s sustained visual attention was preferentially directed toward same-sex targets. Faces served as attentional anchors, attracting both early attention and deeper processing, as reflected in longer fixation durations, whereas gender differences were more pronounced for bodies, with male bodies consistently receiving the least attention. Introspective access was metric-specific: Self-reports aligned most strongly with fixation duration, moderately with first fixation, weakly with total dwell time, and not with fixation count. At the cue level, early orienting showed only limited correspondence with the most salient self-reported cue, but stronger alignment when broader any-match criteria were applied. Self-reports prioritized expressive (e.g., smile, eyes) and grooming facial cues (e.g., hairstyle, beard), as well as sexually dimorphic and stylistic body-related cues (e.g., legs, breasts, outfit, and body shape for women; hands, outfit, shoulder width, and arm muscles for men).

**Conclusion:**

These findings indicate that romantic visual attention operates across distinct processing stages, with only partial overlap between what individuals look at and what they report. While sustained attention is partly accessible to introspection, early attentional processes remain largely automatic, highlighting limits in awareness during initial impression formation.

## 1 Introduction

When encountering a potential romantic partner, where do women actually look—and how closely does looking behavior align with what women believe captured their attention? In general, people form snap judgments about potential partners within milliseconds, using visual shortcuts to assess compatibility, attractiveness, and sociosexual intentions (Bar et al., 2006; Todorov et al., 2015; Willis and Todorov, 2006). These early evaluations originate from automatic cognitive and affective processes that influence subsequent interactions (Finkel et al., 2012). In this context, both facial and body-related visual cues serve as *markers of attractiveness*, influencing perceptions of confidence, approachability, and mating potential (Hall et al., 2010; Langlois et al., 2000; Rhodes, 2006). Although facial cues are often assumed to dominate first impressions, empirical evidence suggests a more nuanced pattern of visual prioritization, with bodies and contextual information also playing an important role (e.g., Alicke et al., 1986; Currie and Little, 2009; Slaughter et al., 2004). Importantly, individuals’ ability to retrospectively report which cues guided these rapid impressions may be limited, as introspective access to early attentional processes is constrained. This study, therefore, investigated the correspondence between measured visual attention (via eye-tracking) and self-reported salience in women’s romantic perception to determine which visual attraction cues dominate early impression formation.

Romantic attraction is not only a matter of subjective preference but also a process shaped by evolutionary pressures and cognitive mechanisms (e.g., Grammer et al., 2003; Little et al., 2011; Thornhill and Gangestad, 1999). Evolutionary theories propose that humans have developed innate preferences for physical traits (e.g., facial symmetry, sexual dimorphism, and averageness) signaling genetic fitness, reproductive potential, and overall health of potential mating partners (e.g., Fink and Penton-Voak, 2002). These preferences are shaped by natural and sexual selection, with facial and body-related attraction cues serving as reliable indicators of mate quality across cultures (Thornhill and Gangestad, 1999). One of the central cues, according to evolutionary psychology, is sexually dimorphic traits—physical characteristics that differ between men and women due to hormonal influences and adaptive reproductive strategies. In male faces, higher testosterone levels contribute to stronger jawlines, pronounced cheekbones, and brow ridges—cues associated with dominance and genetic fitness. Conversely, in female faces, higher estrogen levels result in smoother skin, larger eyes, and fuller lips—cues linked to fertility and reproductive health (Little et al., 2011). According to Rhodes (2006), these sexually dimorphic face markers influence both immediate visual attention and long-term romantic preferences.

Specifically, men, whose reproductive success depends on access to fertile partners, tend to prioritize physical cues that indicate youthfulness and fertility (Buss, 1989). According to evolutionary theory, this explains not only the use of the above-mentioned face cues but also their stronger orienting of visual attention to female waist-to-hip ratio (WHR), breast size, and facial symmetry—all indicators of reproductive health (Singh, 1993). By contrast, women exhibit a dual-focus mating strategy: While short-term attraction may indeed favor masculine cues indicative of genetic fitness, long-term mate selection emphasizes traits linked to resource provision, social status, and emotional commitment (Li et al., 2002). These differences in attraction priorities are also reflected in how men and women visually assess potential partners.

While evolutionary perspectives emphasize innate preferences, romantic attraction is also shaped by sociocultural influences. Cultural beauty standards often inform which visual cues are perceived as attractive, and individuals internalize these standards through media exposure, socialization, and peer reinforcement (Etcoff, 1999; Langlois et al., 2000). Beyond stable features such as faces and bodies, contextual cues—most notably clothing—can further modulate initial attraction judgments. According to self-reports, women believe they notice clothes first for both same- and opposite-sex individuals, while men believe they prioritize body and face for opposite-sex people and clothes for same-sex people, but for members of the opposite sex, clothes take third place (as cited in Knapp et al., 2014, p. 186).

Despite the extensive literature on self-reported attraction preferences (e.g., Buss and Schmitt, 2019; Buyukcan-Tetik et al., 2017; Fink et al., 2015; Fletcher et al., 1999; Li et al., 2002), traditional self-report methods are limited by social desirability bias, memory distortions, and a lack of introspective access. Individuals may not accurately report what captures their visual attention, either because they forget (e.g., during retrospective recall) or remain unaware of their own preferences. This phenomenon—*introspective inaccuracy*—is well-documented in research on social perception and decision-making (Fazio and Olson, 2003; Nisbett and Wilson, 1977). In the domain of attraction, such potential discrepancies between self-reports and actual gaze behavior are especially relevant, as visual preferences are also shaped by implicit, unconscious influences (Eastwick and Finkel, 2008; Jiang et al., 2006). As such, a gap often exists between what individuals think they find attractive and what actually captures their attention in real-time, making eye-tracking an essential tool for capturing priorities of selection mechanisms (e.g., Valuch et al., 2015).

Eye tracking offers a direct and minimally intrusive method for recording gaze allocation across defined areas of interest (AOIs) and is closely linked to attentional capture mechanisms (Deubel and Schneider, 1996; Duchowski, 2017). Recent methodological work further emphasizes that eye-tracking metrics capture distinct components of visual processing rather than a single unitary construct of attention (Hessels et al., 2025), supporting the present multi-metric approach. Unlike self-reports, which are susceptible to introspective bias and *post hoc* reconstruction, eye-tracking provides an objective index of which visual cues attract attention during impression formation (Brône and Oben, 2018; Holmqvist et al., 2011; Valtakari et al., 2021). Moreover, fixation behavior can reflect implicit preference processes, such as mere exposure effects, that operate independently of explicit attraction judgments (e.g., Shimojo et al., 2003). By integrating eye-tracking metrics with self-reported evaluations, researchers can directly assess the correspondence between perceived and measured attentional priorities, thereby offering a more precise characterization of attentional biases in romantic contexts (Tatler et al., 2011). Accordingly, drawing on dual-process models of cognition (e.g., Kahneman, 2011; Slovic et al., 2005), romantic visual attention can be conceptualized as fast, automatic, bottom-up processes (System 1), which operate rapidly and largely outside conscious awareness, and slower, reflective, top-down mechanisms (System 2), which are shaped by social norms, cultural scripts, and identity-relevant interpretations of attractiveness. Within this framework, eye-tracking metrics provide objective, real-time measures of attentional priorities (System 1), whereas self-reports capture *post hoc* interpretations and evaluations of attraction based on memory (System 2).

Eye-tracking research on gender differences in visual attention suggests that men and women allocate attention to social stimuli in systematically different ways, with implications for mate selection and attraction judgments (e.g., Jiang et al., 2006). For example, Lykins et al. (2008) found that when viewing both erotic and non-erotic stimuli, men fixated significantly longer on opposite-sex figures, whereas women distributed their gaze more evenly across opposite- and same-sex figures; notably, no gender differences emerged in attention to contextual background elements. A further gendered divergence became apparent when considering sexually dimorphic traits. Nummenmaa et al. (2012) found that although both men and women initially fixated on faces, subsequent gaze patterns diverged: Men shifted their attention toward women’s breasts, whereas women allocated more attention to men’s pelvic or genital regions. Their findings further showed that nude bodies elicited more extensive visual inspection than clothed figures, and that increased attention to sexually relevant body regions was associated with heightened physiological arousal, indicating a close link between gaze behavior and sexual interest. In line with this, recent eye-tracking research suggests that women’s visual attention to body-related content is shaped not only by perceptual salience but also by socio-cognitive factors linked to appearance evaluation and well-being (Jabłońska, 2024). Beyond facial and body-related cues, background elements in scenes—despite receiving comparatively less attention—have been also shown to contribute to contextual processing and impression formation (Nummenmaa et al., 2012).

Beyond these cue-specific differences, eye-tracking studies also suggest that women tend to adopt a more exploratory viewing strategy than men, characterized by longer scan paths, larger saccade amplitudes, and faster overall inspection of visual scenes (Sargezeh et al., 2019). This broader scanning style implies a more distributed allocation of attention that extends beyond focal targets to include contextual elements. Taken together, these findings underscore the importance of accounting for gender-specific attentional strategies, the interplay between evolutionary and sociocultural influences, and the contribution of both focal and peripheral visual cues when investigating visual attention and preference formation in romantic contexts.

Based on previous findings on introspective inaccuracy (e.g., Eastwick and Finkel, 2008; Fazio and Olson, 2003), we first hypothesized a dissociation between women’s self-reported visual preferences and their measured gaze behavior when viewing romantic first-impression scenes. Specifically, we expected alignment at the broader AOI level (e.g., face, body), but divergence at the cue-specific level (e.g., eyes, smile, beard), reflecting limited introspective access to initial versus overall sustained gaze deployment and/or different weighting of these attentional indices for retrospectively self-reported preferences. Second, we expected that faces would elicit longer fixation durations than body-related or background areas, consistent with their central role in social, emotional, and romantic perception (Cerf et al., 2007; Fink and Penton-Voak, 2002; Henderson et al., 2005; Rhodes, 2006), while acknowledging recent evidence that body-related information also contributes to social evaluation (Morrison and Lanigan, 2025). Third, grounded in findings from Lykins et al. (2008) and Sargezeh et al. (2019), we predicted that visual attention would be distributed relatively evenly between same- and opposite-sex figures. Finally, based on evolutionary psychology and mate selection theories, we predicted that sexually dimorphic traits—such as body shape, chest, and shoulders for male stimuli, and WHR or breast prominence for female stimuli—would attract early fixations, consistent with their proposed role as rapid visual heuristics in attractiveness evaluation (Buss, 1989; Little et al., 2011; Singh, 1993; Thornhill and Gangestad, 1999).

As our testbed, we selected images of heterosexual couples from the first episode of the reality dating show *The Bachelor(ette)*. This segment features contestants meeting the lead for the first time, a moment explicitly designed to attract attention, signal interest, and stand out—making it well-suited for studying early romantic dynamics. Although partially scripted, the corresponding roleplay is inherent to courtship behavior (Givens, 1978; Grammer et al., 2000) and, therefore, still reflects authentic nonverbal cues and visual signals relevant to romantic perception. Moreover, the static nature of the images allowed participants to observe the scenes without fear of reciprocal interaction—a factor known to suppress gaze behavior in live settings (Laidlaw et al., 2011). This likely increased the frequency of socially relevant fixations and created a more sensitive test environment. Additionally, using still images instead of video eliminated motion cues, which are powerful bottom-up attractors of visual attention (Pratt et al., 2010). By removing the distracting effect of motion, we could better observe how participants’ gaze was influenced by theoretically more relevant factors—like social expectations and evolutionary instincts about attractiveness—rather than automatic responses to visual movement. To avoid biasing participants’ gaze behavior, we deliberately withheld the study’s focus on romantic attraction and did not require explicit attractiveness or beauty ratings. This approach ensured that participants remained open-minded rather than task-oriented, allowing for more naturalistic attention patterns. While this design enhances ecological validity, its limitations are addressed in the General Discussion.

## 2 Methods

### 2.1 Participants

Using G-Power 3.1 software, an *a priori* power analysis for a repeated-measures ANOVA (within-subject factor with five measurements) indicated that a total sample size of at least 45 female participants is required to detect a medium effect size (*f* = 0.25) with 80% power (*α* = 0.05), assuming conservative parameters for within-subject designs. As such, 68 heterosexual female participants (age range: 18–29 years, *M* = 23.5, *SD* = 2.9) with normal or corrected-to-normal vision took part in the study. Participants were recruited from the University of Vienna participant pool and received partial course credit in exchange for voluntary participation. All participants provided written informed consent prior to participation and were unaware of the study’s purpose until debriefing. The study was approved by the Ethics Committee of the University of Vienna, and data collection took place in the MediaLab of the University of Vienna.

### 2.2 First impression stimuli

Participants viewed 10 images of heterosexual couples engaged in romantic interactions. All images were presented full-screen and centered on a 2,560 × 1,440 pixels monitor. To ensure eye-tracking accuracy, each image was embedded within a neutral 50% gray background, occupying roughly one-third of the screen, ensuring that participants’ gaze remained within the central high-precision zone. Images were not cropped or resized, preserving original proportions and composition. Brightness levels were standardized to control for luminance-driven attention effects. To reduce side-bias effects, gender placement was counterbalanced: In half of the images, a woman appeared on the left side and a man on the right; in the remaining five images, this was reversed. Each image was displayed for 10 s, followed by a 1-s blank screen to minimize carry-over and after-image effects. To mitigate order-related biases, presentation order was randomized across participants. The visual design and framing across all images followed standardized parameters:

#### 2.2.1 Composition

Couples are seated at close distance, fully visible, centered, and facing slightly toward each other. Their gazes are directed at one another, accompanied by open body language (e.g., smiling, leaning forward), conveying social engagement and mutual interest. Backgrounds featured warm ambient lighting (e.g., fireplaces, candles, soft interior decor) to simulate intimate, emotionally engaging environments, but all remain neutral and non-distracting.

#### 2.2.2 AOIs

Five AOIs were defined per image based on the existing above-discussed findings: (1) female face; (2) male face; (3) female body; (4) male body; (5) background. All AOIs were consistently visible without occlusion, manually drawn using freeform polygons. All the scenes were balanced to contain both partners symmetrically. AOI sizes were well matched: *female faces* (*M* = 7,829 pixels, px, *SD* = 268), *male faces* (*M* = 8,076 px, *SD* = 248), *female bodies* (*M* = 115,783 px, *SD* = 2,558), *male bodies* (*M* = 115,547 px, *SD* = 2,622), and *background* (*M* = 1,234,204 px, *SD* = 6,393). Paired *t*-tests confirmed no significant gender differences in face or body sizes: *t* (9) = −1.80, *p* = 0.11 (*faces*), *t* (9) = 0.23, *p* = 0.82 (*bodies*). Thus, any gaze asymmetries for different genders are unlikely due to AOI size disparities.

Eye movements were recorded using the EyeLink 1000 Plus system (SR Research; RRID: SCR_009602), with the desktop-mounted camera positioned below the display. This system operated in remote, head-free mode, allowing natural viewing at a distance of approximately 70 cm within a 32° × 25° recording area. A 25 mm lens was used in binocular tracking mode, with both eyes recorded at a sampling rate of 500 Hz. To enhance precision without restricting movement, target stickers were placed on participants’ foreheads instead of using a chin rest. Calibration and validation followed a standard nine-point procedure, with an acceptance threshold of <0.5° of visual angle. When this threshold was not met, calibration was repeated until acceptable accuracy was achieved. Gaze data were recorded using SR Research’s proprietary EyeLink software and analyzed with Data Viewer.

Fixations were identified using the EyeLink system’s default velocity- and acceleration-based algorithm (SR Research Ltd., 2023), with standard thresholds (velocity: 30°/s; acceleration: 8,000°/s^2^). A minimum fixation duration threshold of 100 ms was applied to distinguish meaningful fixations from brief oculomotor events, consistent with established practice in eye-tracking research (Holmqvist et al., 2011; Manor and Gordon, 2003). Trials with excessive track loss (i.e., more than 25% missing data within a trial) were excluded from further analysis. Overall, data quality was high, and the proportion of excluded trials was minimal (less than 5% of trials were excluded due to data quality criteria). Remaining data were visually inspected to ensure robustness of fixation classification.

### 2.3 Experimental procedure and data analysis

#### 2.3.1 Measured visual attention (eye-tracking metrics)

To assess how women visually engaged with romantic scenes, we followed Bojko’s (2013, p. 125) framework, focusing on multiple complementary eye-tracking metrics indexing distinct dimensions of visual engagement. Specifically, we examined early orienting, sustained attention, and repeated visual engagement, alongside a fine-grained cue-level analysis within preselected AOIs. Key eye-tracking metrics were extracted:

(1) *Total fixation duration,* also known as *dwell time* (i.e., sustained attention)—the cumulative time (in ms) spent fixating within each AOI. This metric reflects the overall attentional allocation.
(2) *Average fixation duration* (i.e., depth of cognitive engagement)—the mean duration (in ms) of individual fixations per AOI. This metric captures how long attention was maintained during each glance and is commonly interpreted as an index of cognitive processing depth, independent of fixation frequency (Holmqvist et al., 2011).
(3) *Fixation count* (i.e., repeated visual engagement)—the total number of discrete fixations made within each AOI, reflecting recurring visual interest or attentional returns.
(4) *First-fixated AOI* (i.e., immediate visual salience)—the first AOI receiving a meaningful fixation (≥100 ms). To control for central fixation bias, initial fixations landing on the *central* background region were excluded as such fixations likely reflect default oculomotor anchoring rather than purposeful visual engagement (Tatler, 2007).
(5) *First-fixated attraction cue within each AOI* (i.e., fine-grained first gaze)—the first facial (e.g., eyes, nose, lips) or body-related cue (e.g., legs, shoulders, chest) fixated within each AOI, identified by mapping gaze coordinates using a custom Python script.

Before examining correspondence between gaze metrics and self-reports, we evaluated the internal coherence of the gaze measures by computing within-participant Spearman rank-order correlations among total dwell time, average fixation duration, and fixation count.

#### 2.3.2 Perceived visual attention (self-reported preferences)

Following the eye-tracking session, participants completed two self-report tasks via the SoSci Survey platform to assess perceived visual attention at both AOI and cue-specific levels for direct comparison with the corresponding eye-tracking metrics.

(1) *Perceived hierarchy of AOIs*—Participants ranked the five AOIs in order of perceived salience (1 = the most salient, 5 = the least salient). Spearman rank-order correlations were computed between these rankings and gaze metrics (total dwell time, average fixation duration, first fixation, and fixation count). These individual correlation coefficients were then tested against zero using one-sample *t*-tests across participants.
(2) *Perceived attraction cue salience order across AOIs*—Participants listed up to three specific attraction cues (e.g., eyes, hands, legs) for each AOI (excluding *background*) in descending order of perceived salience. Conceptually overlapping cues (e.g., *smile, teeth,* and *lips)* were treated as aligned. To maintain a conservative bias in favor of alignment, coders included any fixation that could reasonably link to a reported cue. To reduce priming, habituation, or fatigue effects, only participants’ *first scene exposure* was analyzed.

To examine correspondence between perceived and measured attentional priorities, two complementary analyses were conducted based on binary match criteria. The first analysis focused on exact alignment, defined as instances in which the first meaningful fixation coincided with the cue participants reported as most salient (Cue 1). For each trial, this was coded as a binary outcome (1 = match, 0 = no match). The second analysis assessed broader alignment, defined as instances in which the first fixation corresponded to any of the three cues reported by participants (Cues 1–3). This measure captures whether early attentional orienting fell within the set of subjectively salient features, regardless of their reported priority. Again, outcomes were coded binarily (1 = match to any reported cue, 0 = no match). Both exact and broader match outcomes were analyzed using *generalized linear mixed-effects models* with a binomial distribution and logit link function. AOI (female face, male face, female body, male body) was included as a fixed effect, and participant was modeled as a random intercept to account for within-subject dependence.

Finally, to examine how perceived salience of attraction cues unfolded across impression formation, we analyzed the order in which participants reported attraction cues within each AOI. Frequency counts were computed for first, second, and third cues to identify whether particular cues were disproportionately emphasized early or late in the reporting sequence, and whether these patterns varied by AOI or target gender. *Temporal salience* was assessed using chi-square goodness-of-fit tests comparing observed mention frequencies against a uniform distribution across mention order. Inferential analyses were conducted only for cues with sufficient expected cell frequencies (≥5). This approach provides insight into participants’ internal models of which visual attraction cues are perceived as most salient during romantic first-impression formation.

## 3 Results

### 3.1 Total dwell time (sustained attention)

Total dwell time was analyzed across 10 scenes to assess sustained visual engagement with each AOI. This metric reflects how long participants allocated attention to a given region overall. As shown in Table 1, *female faces* received the longest dwell time (*M* = 2,174 ms, *SD* = 981), followed by *male faces* (*M* = 1,854 ms, *SD* = 783), *female bodies* (*M* = 1,721 ms, *SD* = 683), *background* (*M* = 1,537 ms, *SD* = 885), and *male bodies* (*M* = 1,268 ms, *SD* = 568). A repeated-measures analysis of variance (ANOVA) revealed a statistically significant effect of AOI on total dwell time, *F* (4, 268) = 10.52, *p* < 0.001, *η*^2^*ₚ* = 0.13. Bonferroni-corrected *post hoc* comparisons revealed that *female faces*, *male faces*, and *female bodies* all received significantly more dwell time than *male bodies* (all *p*s < 0.001; *d*s = 0.57–1.14). No other comparisons reached significance (*p*s ≥ 0.024). To further examine attentional allocation by target gender, AOIs were grouped into same-sex (*female face* + *female body*) and opposite-sex (*male face* + *male body*) categories. Same-sex AOIs elicited significantly greater sustained visual attention than opposite-sex AOIs, *t* (67) = 4.61, *p* < 0.001, *d* = 0.56 (Cohen, 1988), indicating a medium-sized effect.

**Table 1:** Comparative rankings of women’s measured visual attention and self-reported salience across areas of interest in romantic first impressions.

AOI	Fixation duration (ms, across 10 scenes)				Total fixation count (*n*, across 10 scenes)		First fixation (≥100 ms, across 10 scenes)		Self-reported AOI salience	
	Total dwell time	Ranking	Average fixation duration	Ranking	Fixation count	Ranking	First fixation (*n*, % of trials)	Ranking	Mean of perceived AOI rank	Ranking
Female face	2,174	1st	434	1st	49	3rd	282 (42%)	1st	1.57	1st
Male face	1,854	2nd	398	2nd	44	5th (last)	123 (18%)	3rd	2.03	2nd
Female body	1,721	3rd	289	3rd	59	2nd	129 (19%)	2nd	2.97	3rd
Male body	1,268	5th (last)	267	4th	46	4th	101 (15%)	4th	3.68	4th
Background	1,537	4th	242	5th (last)	62	1st	36 (5%)	5th (last)	4.75	5th (last)

### 3.2 Average fixation duration (depth of cognitive engagement)

Average fixation duration was examined to assess how long visual attention was sustained once an AOI had been selected, providing an index of cognitive engagement depth within each AOI. *Female faces* showed the longest average fixation duration (*M* = 434 ms, *SD* = 148), followed by *male faces* (*M* = 398 ms, *SD* = 127), *female bodies* (*M* = 289 ms, *SD* = 62), *male bodies* (*M* = 267 ms, *SD* = 62), and background (*M* = 242 ms, *SD* = 41). A repeated-measures ANOVA revealed a significant main effect of AOI, *F* (4, 268) = 74.58, *p* < 0.001, *η*^2^*ₚ* = 0.53. Bonferroni-corrected *post hoc* comparisons showed that *female faces* were fixated longer than *female bodies*, *male bodies*, and *background* (all *p*s < 0.001; *d*s = 1.50–1.97), but did not differ from *male faces* (*p* = 0.269). In addition, *male faces* and *female bodies* were both fixated longer than *male bodies* (all *p*s < 0.001; *d*s = 1.31–1.34), and *female bodies* were also fixated longer than *background* (*p* < 0.001; *d* = 0.48). No other comparisons reached significance.

### 3.3 Fixation count (repeated visual engagement)

Fixation count was analyzed as an index of repeated visual engagement, reflecting the total number of discrete fixations irrespective of fixation duration made within each AOI, and regardless of how many times participants entered or exited that area. *Background* elicited the highest number of fixations (aggregated across 10 scenes; *M* = 62, *SD* = 35), followed by *female bodies* (*M* = 59, *SD* = 24), *female faces* (*M* = 49, *SD* = 21), *male bodies* (*M* = 46, *SD* = 21), and *male faces* (*M* = 44, *SD* = 20). A repeated-measures ANOVA revealed a significant main effect of AOI, *F* (4, 268) = 7.85, *p* < 0.001, *η*^2^*ₚ* = 0.11. Bonferroni-corrected post hoc comparisons showed that *female bodies* were fixated more often than both *male faces* (*p* = 0.002; *d* = 0.60) and *male bodies* (*p* < 0.001; *d* = 0.53), and that *background* received more fixations than *male bodies* (*p* = 0.003; *d* = 0.65). No other comparisons reached significance (*p*s ≥ 0.015).

### 3.4 First-fixated area of interest (AOI) (immediate visual salience)

To examine immediate visual salience, we analyzed the first AOI to receive a meaningful fixation (≥100 ms) during scene viewing. Across 10 scenes (68 participants, 680 trials), 671 valid first fixations were retained after applying the meaningful fixation criterion. Observed frequencies differed significantly from a uniform distribution, *χ*^2^ (4) = 244.00, *p* < 0.001, indicating systematic differences in early attentional allocation. First fixations most frequently landed on *female faces* (*n* = 282; 42%), followed by *female bodies* (*n* = 129; 19%), *male faces* (*n* = 123; 18%), *male bodies* (*n* = 101; 15%), and *background* (*n* = 36; 5%). First fixation duration was additionally examined as an index of early processing depth. Fixations on faces were longer than those on bodies and background, with the longest durations observed for *female faces* (*M* = 334 ms, *SD* = 251) and *male faces* (*M* = 331 ms, *SD* = 272), followed by *male bodies* (*M* = 222 ms, *SD* = 131), *female bodies* (*M* = 216 ms, *SD* = 82), and *background* (*M* = 198 ms, *SD* = 73). Effect size estimates indicated moderate-to-large differences for contrasts involving facial versus non-facial regions (e.g., faces vs. background, *d*s = 0.67–0.74; faces vs. bodies, *d*s = 0.51–0.63). In contrast, differences among body regions and between body and background areas were small (*d*s = 0.06–0.23), as were differences between female and male faces (*d* = 0.01).

### 3.5 Self-reported AOI salience

Women’s self-reported salience ratings revealed a strongly differentiated hierarchy across AOIs. *Female faces* were rated as the most salient visual targets (*M* = 1.57, *SD* = 0.72), followed by *male faces* (*M* = 2.03, *SD* = 0.96), *female bodies* (*M* = 2.97, *SD* = 0.95), *male bodies* (*M* = 3.68, *SD* = 0.91), and *background* regions (*M* = 4.75, *SD* = 0.63). A repeated-measures ANOVA indicated a significant effect of AOI, *F* (4, 268) = 124.30, *p* < 0.001, *η*^2^*ₚ* = 0.65. Bonferroni-corrected *post hoc* comparisons indicated that *female faces* were ranked as more salient than all other AOIs (all *p*s < 0.001), except for *male faces* (*p* = 0.111). *Male faces* were ranked as more salient than both *body regions* and *background* (all *p*s ≤ 0.007), and *female bodies* were ranked higher than *background* (*p* < 0.001). The difference between *female* and *male bodies* was not significant (*p* = 0.007). Effect sizes indicated substantial perceived differences across AOIs, particularly for contrasts involving *background* and *male bodies* (*d*s = 1.27–3.77). Differences between *facial* and *body AOI* were also large (*d*s = 0.84–1.66), whereas the contrast between *female* and *male faces* was comparatively small and not statistically significant (*d* = 0.54).

### 3.6 Relationships among gaze metrics and correspondence with self-reports

Before examining correspondence with self-reports, we assessed the internal relationships among gaze metrics using within-participant Spearman correlations tested against zero with one-sample *t*-tests. *Total dwell time* and *fixation count* were strongly and positively correlated (*Mρ* = 0.68, *SD* = 0.37), *t* (67) = 15.06, *p* < 0.001, indicating that both metrics captured a shared dimension of overall visual engagement. *Total dwell time* was also moderately correlated with *average fixation duration* (*Mρ* = 0.51, *SD* = 0.43), *t* (67) = 9.85, *p* < 0.001, suggesting partial overlap between cumulative viewing time and fixation depth. In contrast, the association between *average fixation duration* and *fixation count* was weak and not statistically significant (*Mρ* = 0.08, *SD* = 0.56), *t* (67) = 1.15, *p* = 0.25, indicating that fixation duration and fixation frequency reflected largely independent aspects of gaze behavior.

We next examined the correspondence between gaze metrics and self-reported AOI salience using the same analytical approach. Correlations between *total dwell time* and *self-reported salience* were small but significantly positive (*Mρ* = 0.25, *SD* = 0.57), *t* (67) = 3.60, *p* < 0.001, indicating modest alignment between cumulative viewing time and explicit evaluations. A substantially stronger correspondence emerged for *average fixation duration* (*Mρ* = 0.58, *SD* = 0.41), *t* (67) = 11.85, *p* < 0.001, suggesting that participants were more sensitive to regions that elicited longer, more sustained fixations. Correlations based on *first fixation* were also positive (*Mρ* = 0.43, *SD* = 0.50), *t* (67) = 7.07, *p* < 0.001, indicating that early attentional orienting was partially reflected in self-reports. In contrast, *fixation count* was not significantly associated with *self-reported salience* (*Mρ* = −0.07, *SD* = 0.52), *t* (67) = −1.06, *p* = 0.293, indicating that the frequency of gaze shifts across AOIs was not reliably captured by participants’ explicit judgments.

### 3.7 Measured versus self-reported attraction cue salience

To examine how closely women’s early visual attention aligned with their introspective impressions of attraction cues, we compared first-fixated cues with self-reported cue salience across four AOIs: *female face*, *female body*, *male face*, and *male body*. For each AOI, participants listed up to three attraction cues in descending order of perceived importance.

#### 3.7.1 Alignment between first fixation and self-reported cues: exact and any-match analyses

To test whether early attentional orienting aligned with self-reported cue salience, a generalized linear mixed-effects model examined whether the first fixation matched the participant’s most salient self-reported cue (Exact Match). Match probability differed significantly across AOIs, *χ*^2^ (3) = 10.50, *p* = 0.015 (Table 2). Estimated marginal means indicated that exact matches were most likely for *female faces* (*M* = 0.28, 95% CI [0.18, 0.41]), followed by *male faces* (*M* = 0.20, 95% CI [0.12, 0.33]) and *male bodies* (*M* = 0.19, 95% CI [0.11, 0.31]), with the lowest probability observed for *female bodies* (*M* = 0.08, 95% CI [0.03, 0.17]). A second model assessed broader correspondence by testing whether the first fixation matched any of the three reported cues (Any Match). This analysis again revealed a significant main effect of AOI, *χ*^2^ (3) = 21.15, *p* < 0.001. Estimated marginal means showed that matches were most likely for *female faces* (*M* = 0.69, 95% CI [0.56, 0.80]), followed by *male bodies* (*M* = 0.50, 95% CI [0.37, 0.63]) and *male faces* (*M* = 0.39, 95% CI [0.27, 0.52]), whereas *female bodies* showed the lowest match probability (*M* = 0.31, 95% CI [0.20, 0.44]).

**Table 2:** Alignment between measured first fixation and self-reported attraction cues across areas of interest (generalized linear mixed models).

AOI	Estimated probability	SE	95% CI
Exact match (measured first fixation = self-reported cue 1)			
Female face	0.28	0.06	[0.18, 0.41]
Male face	0.20	0.05	[0.12, 0.33]
Male body	0.19	0.05	[0.11, 0.31]
Female body	0.08	0.03	[0.03, 0.17]
Model effect: *χ*^2^ (3) = 10.50, *p* = 0.015			
Any match (measured first fixation = self-reported cues 1–3)			
Female face	0.69	0.06	[0.56, 0.80]
Male body	0.50	0.07	[0.37, 0.63]
Male face	0.39	0.07	[0.27, 0.52]
Female body	0.31	0.06	[0.20, 0.44]
Model effect: *χ*^2^ (3) = 21.15, *p* < 0.001			

Estimates represent predicted probabilities from generalized linear mixed-effects models with binomial family and logit link. Confidence intervals are based on model estimates.

#### 3.7.2 Temporal prioritization of attraction cues within AOIs

To examine whether specific attraction cues were emphasized earlier or later during self-reporting, chi-square goodness-of-fit tests compared observed cue frequencies across mention positions (first, second, third) against a uniform distribution. Only cues meeting the minimum expected cell frequency criterion (≥5) were included in inferential analyses.

*Female face*: A total of nine visual attraction cues were reported for *female faces*: *eyes*, *smile*, *hairstyle*, *makeup*, *cheekbones*, *nose*, *eyebrows*, *dimples*, and *freckles*. The most frequently mentioned cues were *smile* (*n* = 65, 32%), *eyes* (*n* = 61, 30%), and *hairstyle* (*n* = 52, 25%). All the remaining cues occurred only sporadically (each ≤6%) and were therefore excluded from inferential analyses due to insufficient expected frequency. Chi-square goodness-of-fit tests revealed cue-specific temporal patterns. *Smile* showed the strongest deviation from a uniform distribution across mention positions, *χ*^2^ (2) = 16.99, *p* < 0.001, indicating pronounced early salience (Cue 1: *n* = 36; Cue 2: *n* = 20; Cue 3: *n* = 9). *Hairstyle* also deviated significantly, *χ*^2^ (2) = 9.98, *p* = 0.007, but in the opposite direction, reflecting increased salience in later mentions (Cue 1: *n* = 7; Cue 2: *n* = 20; Cue 3: *n* = 25). In contrast, mentions of *eyes* did not differ significantly from a uniform distribution, *χ*^2^ (2) = 2.2, *p* = 0.33, suggesting relatively stable salience across reporting positions.

*Male face*: Ten cues were reported for *male faces*: *smile*, *beard*, *hairstyle*, *eyes*, *jawline, clean-shaven face, nose, cheekbones, eyebrows,* and *glasses*. Among these, *smile* (*n* = 53, 26%), *beard* (*n* = 42, 21%), *hairstyle* (*n* = 41, 20%), *eyes* (*n* = 38, 19%), and *jawline* (*n* = 15, 7%) accounted for the majority of mentions. All the remaining cues each accounted for ≤2% of total mentions and were excluded from inferential analyses. Chi-square goodness-of-fit tests revealed no significant deviations from a uniform distribution for the core male-face cues: *smile*, *χ*^2^ (2) = 1.40, *p* = 0.50; *beard*, *χ*^2^ (2) = 0.43, *p* = 0.81; *hairstyle*, *χ*^2^ (2) = 0.63, *p* = 0.73; *eyes*, *χ*^2^ (2) = 2.26, *p* = 0.32; and *jawline*, *χ*^2^ (2) = 2.8, *p* = 0.25. Together, these results indicate that—unlike female-face cues—*male-face features* were not temporally prioritized within self-reports.

*Female body*: A total of 14 visual attraction cues were reported for *female bodies*: *breasts*, *legs*, *outfit*, *body shape*, *hands*, *accessories*, *neckline*, *shoulders*, *arms*, *shoes*, *nails*, *height*, *muscles*, and *waist*. The most frequently mentioned cues were *breasts* (*n* = 40, 20%), *legs* (*n* = 40, 20%), and *outfit* (*n* = 37, 18%), followed by *body shape* (*n* = 28, 14%), and *hands* (*n* = 19, 9%). All remaining cues each accounted for ≤7% of mentions and were excluded from inferential analyses. Chi-square goodness-of-fit tests revealed cue-specific temporal biases. *Outfit* showed a strong deviation from a uniform distribution, *χ*^2^ (2) = 14.0, *p* < 0.001, indicating pronounced early salience (Cue 1: *n* = 23; Cue 2: *n* = 8; Cue 3: *n* = 6). *Body shape* also deviated significantly, *χ*^2^ (2) = 9.07, *p* = 0.01, reflecting mid-stage emphasis (Cue 1: *n* = 3; Cue 2: *n* = 16; Cue 3: *n* = 9). In contrast, *breasts*, *χ*^2^ (2) = 5.15, *p* = 0.08; *legs*, *χ*^2^ (2) = 1.55, *p* = 0.46; and *hands*, *χ*^2^ (2) = 1.68, *p* = 0.43, did not differ from a uniform distribution, indicating relatively stable salience across reporting positions.

*Male body*: Twelve cues were reported for *male bodies*: *hands*, *outfit*, *(arm) muscles*, *chest*, *shoulder (width)*, *height*, *tattoos*, *torso*, *legs*, *shoes*, *accessories*, and *posture*. The most frequently mentioned cues were *hands* (*n* = 46, 23%), *(arm) muscles* (*n* = 42, 21%), and *outfit* (*n* = 32, 16%), followed by *chest* (*n* = 19, 9%) and *shoulder (width)* (*n* = 19, 9%). All remaining cues each accounted for ≤6% of total mentions and were excluded from inferential analyses. Chi-square goodness-of-fit tests revealed cue-specific temporal biases. *Outfit* showed a strong deviation from a uniform distribution, *χ*^2^ (2) = 13.94, *p* = 0.001, indicating pronounced early salience (Cue 1: *n* = 20; Cue 2: *n* = 9; Cue 3: *n* = 3). *(Arm) muscles* deviated even more strongly from uniformity, *χ*^2^ (2) = 55.10, *p* < 0.001, but in the opposite direction, with mentions increasing sharply across positions (Cue 1: *n* = 3; Cue 2: *n* = 16; Cue 3: *n* = 23). In contrast, *hands*, *χ*^2^ (2) = 4.87, *p* = 0.09; *chest*, *χ*^2^ (2) = 1.37, *p* = 0.50; and *shoulder (width)*, *χ*^2^ (2) = 2.0, *p* = 0.37, showed no significant temporal bias.

## 4 Discussion

### 4.1 Dual-process model of romantic visual attention

The observed convergence and divergence between women’s self-reported attentional priorities and eye-tracking metrics during romantic first-impression formation align with dual-process models of cognition (e.g., Kahneman, 2011; Slovic et al., 2005). Specifically, self-reported AOI salience showed the strongest correspondence with average fixation duration, a weaker but significant association with total dwell time, and a moderate correspondence with first fixation, whereas fixation count was not significantly related to self-reports. This metric-specific gradient indicates that reflective evaluations map most closely onto deeper, sustained processing, while more exploratory scanning behavior remains largely outside conscious awareness. At the cue level, early attentional orienting showed limited alignment with the most salient self-reported cue (Exact Match), with relatively low probabilities across AOIs, but substantially stronger correspondence when a broader Any-Match criterion was applied. This pattern indicates that participants were not reliably aware of the exact location of their initial fixation, but were more likely to recognize whether their gaze fell within a generally relevant cue category. Together, these findings suggest that awareness of attentional allocation is partial and metric-dependent, with higher correspondence for sustained processing and more flexible cue-level alignment than for precise early orienting (Jiang et al., 2006; Nummenmaa et al., 2012).

This dissociation supports the view that early attentional capture during romantic perception is driven primarily by automatic perceptual mechanisms, such as visual salience or stimulus-driven prominence, rather than by conscious intentions or socially shaped preference narratives (e.g., Donk and Van Zoest, 2008). In this regard, our data echo longstanding critiques of relying exclusively on self-reports to infer early attentional processes (Nisbett and Wilson, 1977) and reinforce the value of combining eye-tracking with retrospective measures to demonstrate how automatic and reflective components of romantic attention can be empirically disentangled. In sum, the findings support a dual-process model of romantic visual attention in which automatic perceptual processes (captured by early fixations) operate partly independently from later, conscious appraisal processes or self-reports (captured by self-reported preferences and ranked salience). Eye-tracking not only revealed where women looked, but also when and how these gaze patterns deviated from their own narratives of attraction—offering a fine-grained, time-sensitive map of how romantic impressions unfold from initial perception to reflective judgment.

### 4.2 Metric-specific patterns of visual attention and introspective salience

Total dwell time showed a same-sex bias: Women devoted more sustained viewing to same-sex AOIs (female face + female body) than to opposite-sex AOIs (male face + male body). However, this same-sex effect was not uniform across all gaze metrics. In particular, early fixation duration did not differentiate *female* versus *male faces*, suggesting that the face advantage reflects general social relevance rather than target-gender specificity at the earliest stage of engagement. In contrast, target-gender asymmetry was more pronounced for bodies than for faces. *Female bodies* attracted more sustained attention than *male bodies* in total dwell time and were fixated more frequently than *male bodies*, consistent with the view that women’s body-related attention was more selective and potentially shaped by evaluative goals during impression formation.

Self-reports revealed a strongly differentiated hierarchy. Women retrospectively rated *female faces* as most salient, followed by *male faces*, *female bodies*, *male bodies*, and *background*, with large standardized differences particularly between *facial* and *non-facial regions*, and a small, non-significant advantage of *female* over *male faces*. This explicit ordering is compatible with same-sex attentional biases that may reflect overlapping mechanisms: (1) social comparison processes (Blechert et al., 2009; Festinger, 1954; Sargezeh et al., 2019); (2) sensitivity to socially relevant facial cues (Vanston and Strother, 2017); and/or (3) vigilance toward potential same-gender rivals in mating contexts, as proposed in evolutionary frameworks (e.g., Buss, 1989). While our data cannot definitively disentangle between these explanations or motivations, it underscores the layered nature of visual priorities in romantic first-impression formation—where same-sex stimuli may simultaneously fulfill affiliative, comparative, and competitive roles.

Importantly, women’s attention was organized around both *female* and *male faces* as primary anchors—receiving the highest total dwell time and longest average fixation durations; first-fixation durations were also longer on *faces* than on *bodies* or *background*. This convergence across early orienting, sustained viewing, and fixation depth indicates that facial cues were not only prioritized at the onset of viewing but also elicited deeper and more prolonged processing. This pattern aligns with the central role of faces—particularly expressive facial features—in social evaluation and affective appraisal (e.g., Cerf et al., 2007; Holmqvist et al., 2011), and with the established importance of facial cues in attraction-related judgments (Fink and Penton-Voak, 2002; Rhodes, 2006).

Background regions showed a distinct pattern: although rated as least salient, they elicited the highest fixation counts, indicating frequent but brief attentional returns rather than sustained engagement. This combination likely reflects peripheral monitoring of spatial or contextual features rather than focused engagement and/or active filtering out of these task-irrelevant-looking behaviors by our participants (e.g., Nummenmaa et al., 2012). In general, scanning of the context aligns well with scene perception models, which emphasize the role of early contextual processing in guiding object recognition and/or subsequent eye movements (e.g., Bar, 2007; Castelhano and Henderson, 2007; Oliva and Torralba, 2006; Schyns and Oliva, 1994; Torralba et al., 2006). Thus, fixation count and fixation duration appear to tap into distinct visual functions—fleeting spatial anchoring versus sustained cognitive interest—reinforcing the value of treating these metrics separately when interpreting selection preferences and gaze behavior in attraction research.

Women’s self-reports revealed two dominant attentional themes in *facial attraction cues*: Emotional expressiveness (e.g., *smile*, *eyes*) and grooming features (e.g., *hairstyle*, *beard*, *makeup*). For both male and female faces, expressive cues were consistently prominent across mention positions, confirming their importance in interpersonal evaluation (Hall et al., 2010; Rhodes, 2006). Grooming cues—especially *hairstyle* (for both genders) and *beard* (for male faces)—also appeared frequently. This challenges the assumption that grooming cues are secondary to emotional expressiveness and instead suggests that such stylistic or identity-defining elements are often integrated into initial preference judgments (Clark et al., 1999). Previous research has shown that grooming cues like *hairstyle* and *facial hair* influence social perceptions such as dominance, maturity, and attractiveness—often operating as rapid visual heuristics in mate evaluation (DeBruine et al., 2010; Dixson and Brooks, 2013). The simultaneous self-reported salience of expressive cues and grooming features points to a layered attentional architecture, in which relational signals (Bruce and Young, 1986) and self-presentation markers are cognitively integrated to construct early-stage impressions of preference in romantic scenes.

Body-related self-reports showed a similarly layered pattern. For female bodies, *breasts*, *legs*, and *outfit* were the dominant attentional cues, with *body shape*, *hands*, and *accessories* gaining lower self-reported salience—suggesting a blend of stylistic, sexualized focus with femininity markers. For male bodies, *hands*, *(arm) muscles*, and *outfit* dominated, followed by *chest* and *shoulder (width)*. Notably, several of these cues, such as *breasts*, *(arm) muscles*, *shoulder (width)*, and *body shape*, are also sexually dimorphic and theorized in evolutionary psychology to signal reproductive health, strength, and genetic fitness (Buss, 1989; Singh, 1993). The fact that they appeared consistently across all mention positions supports the idea that such cues are embedded in women’s preference judgments and conscious deliberations of mate value. Together, these findings point to a hybrid attentional model: Women’s self-reported conscious evaluations blend biologically salient traits with socially constructed markers of identity, grooming, and style. Attention unfolds dynamically—from immediate relational signals (e.g., smile, hands) to more symbolic or stylistic features (e.g., tattoos, accessories)—aligning with models that view perception as a wave-like process shaped by both visual bottom-up salience and top-down meaning-making (Tatler et al., 2011; Unema et al., 2005).

### 4.3 Implications, limitations, and future directions

Theoretically, our findings advocate a multimodal approach in future romantic attraction research—one that integrates both the immediacy of gaze and the reflective nature of self-reports. Such integration promises a more nuanced understanding of how romantic first impressions are formed, evaluated, and later articulated. Future research should more explicitly examine the conditions under which alignment between visual attention and self-reported preferences increases or decreases. In particular, individual differences—such as sociosexual orientation, relationship goals, or visual literacy—may systematically moderate the extent to which observers can accurately introspect their own attentional patterns. In addition, the use of different stimulus types (e.g., dynamic interactions, real-life encounters, or culturally diverse contexts) may influence how closely early attentional processes align with later evaluative judgments. Finally, experimental manipulations of task framing (e.g., explicitly romantic vs. neutral viewing conditions) could further clarify the extent to which attentional allocation reflects general social perception versus attraction-specific processing.

But beyond its theoretical contributions, the findings have direct applications in various real-world domains. Dating platforms, for example, rely heavily on vision-based user interactions. Although facial regions received the greatest sustained attention and were retrospectively rated as most salient, body-related cues also attracted early and repeated visual engagement, despite being less prominently acknowledged in self-reported salience. This dissociation suggests that attraction is not exclusively face-driven and that users may underrecognize the role of body-related information in shaping their initial impressions. Incorporating both facial and full-body images may therefore better reflect actual visual behavior during early evaluation.

More broadly, the observed discrepancies between early gaze behavior and self-reported preferences underscore the limits of introspection and suggest that machine learning models and gaze-prediction algorithms—commonly used in UX design, marketing, and dating apps—should integrate implicit eye-tracking data alongside explicit user input for more accurate user profiling. Integrating these data streams could enable the development of hybrid predictive models that more accurately capture user preferences by combining implicit indicators of attention (e.g., gaze patterns) with explicit evaluations. Such models may improve the prediction of visual engagement, support more individualized user profiling, and enhance recommendation systems by accounting for the systematic gap between what users attend to and what they consciously report. In this way, the present study contributes to a process-based understanding of romantic attention by demonstrating that early visual selection and later subjective evaluation are partially dissociable components of impression formation, rather than interchangeable indicators of preference.

Despite its methodological strengths—particularly the integration of eye-tracking with self-reported preferences—our study has several limitations. First, the use of static images, while allowing precise control over visual stimuli, may not fully capture the dynamic and reciprocal nature of real-life romantic encounters. Prior research suggests individuals exhibit greater visual restraint during live social interactions (Laidlaw et al., 2011), implying that absolute levels of face and body fixation observed in laboratory settings may differ from those in naturalistic contexts. Future studies should incorporate dynamic stimuli or interactive paradigms to assess whether the relative attentional patterns observed here generalize to more ecologically valid settings.

Second, the study consisted exclusively of heterosexual female participants, limiting generalizability. Given documented gender and (sexual) orientation-based differences in visual attention (Dixson et al., 2011; Lykins et al., 2008), future research should include more diverse samples, including male, non-binary, LGBTQ+, neurodivergent, and cross-cultural populations. Finally, to avoid priming effects, participants were not explicitly informed that the scenes depicted romantic first encounters. While this approach preserved spontaneous gaze behavior, it may have introduced interpretive ambiguity, as observed gaze patterns could have partly reflected general social perception rather than explicit romantic interest. Future work could systematically compare explicitly framed romantic tasks with neutral viewing conditions to examine how contextual framing modulates both early attentional capture and reflective evaluations.

## Data Availability

The datasets presented in this study can be found in online repositories. The names of the repository/repositories and accession number(s) can be found in the article/supplementary material.
